# Chronic treatment with agomelatine or venlafaxine reduces depolarization-evoked glutamate release from hippocampal synaptosomes

**DOI:** 10.1186/1471-2202-14-75

**Published:** 2013-07-29

**Authors:** Marco Milanese, Daniela Tardito, Laura Musazzi, Giulia Treccani, Alessandra Mallei, Tiziana Bonifacino, Cecilia Gabriel, Elisabeth Mocaer, Giorgio Racagni, Maurizio Popoli, Giambattista Bonanno

**Affiliations:** 1 Department of Pharmacy, Unit of Pharmacology and Toxicology and Center of Excellence for Biomedical Research, Università degli Studi di Genova, Genova, Italy; 2 Laboratory of Neuropsychopharmacology and Functional Neurogenomics, Dipartimento di Scienze Farmacologiche e Biomolecolari and Center of Excellence on Neurodegenerative Diseases (CEND), Università degli Studi di Milano, Via Balzaretti, Milano, 9-20133, Italy; 3 Institut de Recherches Internationales Servier (I.R.I.S.), Suresnes, France; 4 Istituto di Ricovero e Cura a Carattere Scientifico San Giovanni di Dio - Fatebenefratelli, Brescia, Italy

**Keywords:** Antidepressant, Agomelatine, Venlafaxine, Glutamate, Synaptosomes, Hippocampus

## Abstract

**Background:**

Growing compelling evidence from clinical and preclinical studies has demonstrated the primary role of alterations of glutamatergic transmission in cortical and limbic areas in the pathophysiology of mood disorders. Chronic antidepressants have been shown to dampen endogenous glutamate release from rat hippocampal synaptic terminals and to prevent the marked increase of glutamate overflow induced by acute behavioral stress in frontal/prefrontal cortex. Agomelatine, a new antidepressant endowed with MT1/MT2 agonist and 5-HT_2C_ serotonergic antagonist properties, has shown efficacy at both preclinical and clinical levels.

**Results:**

Chronic treatment with agomelatine, or with the reference drug venlafaxine, induced a marked decrease of depolarization-evoked endogenous glutamate release from purified hippocampal synaptic terminals in superfusion. No changes were observed in GABA release. This effect was accompanied by reduced accumulation of SNARE protein complexes, the key molecular effector of vesicle docking, priming and fusion at presynaptic membranes.

**Conclusions:**

Our data suggest that the novel antidepressant agomelatine share with other classes of antidepressants the ability to modulate glutamatergic transmission in hippocampus. Its action seems to be mediated by molecular mechanisms located on the presynaptic membrane and related with the size of the vesicle pool ready for release.

## Background

Compelling evidence suggest that long-term changes in different brain areas and circuits mediating cognitive and emotional behaviors represent the biological underpinnings of mood and anxiety disorders [1-3]. Since the vast majority of neurons and synapses in these areas use glutamate as neurotransmitter [4,5], the glutamatergic system plays a central role in the pathogenesis of different psychiatric disorders and, potentially, appears to be a final common pathway for the therapeutic action of antidepressant agents [3,6-9]. A number of studies have shown that traditional antidepressants regulate ionotropic and metabotropic glutamate receptors and modulate glutamate release and transmission in relevant limbic and cortical areas (for a discussion, see Ref. [10]). In this context, several lines of preclinical/clinical research, which have investigated the action of molecules that directly target the glutamate synapse, are opening the way for glutamatergic, rapid acting, novel antidepressants [6,7].

We have previously found that chronic treatment with different antidepressants (fluoxetine, desipramine, reboxetine) significantly reduced depolarization-evoked glutamate release from hippocampal synaptic terminals (synaptosomes) [11]. The above effect was accounted for by changes in protein phosphorylation [12], in turn affecting protein/protein interactions that regulate the assembly of the presynaptic soluble N-ethylmaleimide-sensitive fusion protein attachment protein receptor (SNARE) complex, mediating fusion of synaptic vesicles with presynaptic membrane [11,13]. We have also shown that acute footshock-stress induces a marked increase of depolarization-evoked overflow of glutamate from prefrontal and frontal cortex synaptosomes, via glucocorticoid receptor activation and SNARE complex accumulation in synaptic membranes. Chronic fluoxetine, desipramine, venlafaxine or agomelatine completely prevented the increase of glutamate release induced by stress [14].

Agomelatine is a new antidepressant, synergically acting as an agonist of MT1/MT2 receptors and as an antagonist of 5-HT_2C_ receptors [15-19]. Although the effects of agomelatine on glutamate release in prefrontal and frontal cortex have been investigated, no data are available on the effects of this drug in hippocampus. Thus, aims of the present work were: (1) To assess whether chronic treatment with agomelatine modulates glutamate release from superfused hippocampal synaptosomes. (2) To study whether agomelatine reduces the accumulation of the presynaptic SNARE complex in hippocampus.

## Results

### Chronic treatment with agomelatine or venlafaxine reduces depolarization-evoked release of glutamate from hippocampal synaptosomes

**Figure 1:**
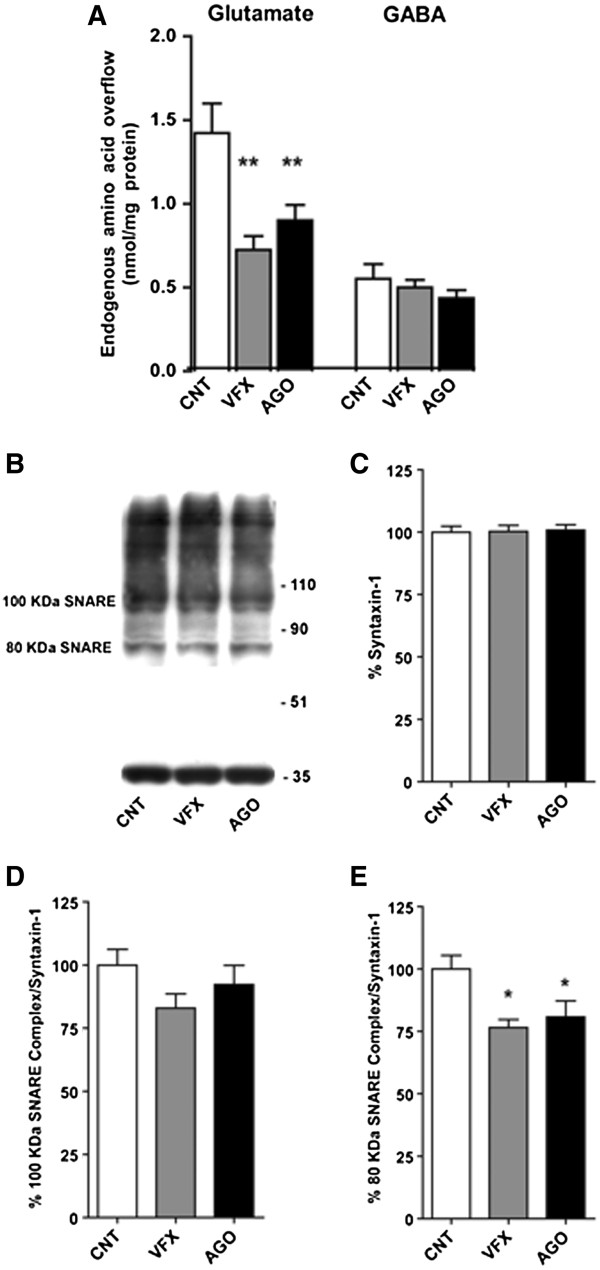
**Effect of chronic treatment with agomelatine or venlafaxine on depolarization-evoked glutamate and GABA release and on SNARE complex accumulation in hippocampal synaptosomes. A)** 15 mM KCl-evoked glutamate and GABA release and 0.5 mM ionomycin-evoked glutamate release from hippocampal synaptosomes of rats chronically treated with vehicle (CNT), agomelatine (AGO) or venlafaxine (VFX). **B)** Representative Western Blot of SNARE complexes in hippocampal synaptosomes visualized with a monoclonal antibody for syntaxin-1. The two SNARE complexes at approximately 100 and 80 kDa and syntaxin-1 monomer are indicated by arrows. Each single SNARE complex was normalized on monomeric syntaxin-1 in the same lane. **C)** Quantitation of syntaxin-1 in CNT and rats chronically treated with VFX or AGO. **D)** Quantitation of normalized 100 kDa SNARE complex in CNT rats and rats chronically treated with VFX or AGO. **E)** Quantitation of normalized 80 kDa SNARE complex in CNT rats, and rats chronically treated with VFX or AGO. All data are expressed as mean ± SEM. * p<0.05, ** p<0.01 vs. CNT rats, Newman-Keuls post-hoc tests following one-way ANOVA (n = 6–10 rats/group in duplicate).

Synaptosomes were purified from the hippocampus of chronically drug- or vehicle-treated rats, and exposed in superfusion to KCl or ionomycin to assess glutamate and γ-amminobutyric acid (GABA) release. As shown in Figure 1A, chronic treatment for 3 weeks with either agomelatine or venlafaxine modified the depolarization-evoked overflow of glutamate (total release minus basal outflow). Indeed, both agomelatine and venlafaxine significantly reduced (F_2,16_ = 8.55, p < 0.01) the 15 mM KCl-induced glutamate overflow (-38% and 49%, respectively). Interestingly, the release of glutamate induced by the calcium ionophore ionomycin (0.5 μM), which mobilizes vesicles from both the recycling and reserve pools (see [11]), was unaffected by drug treatments, suggesting that the action of the drugs is mainly confined to vesicles docked to the presynaptic membrane and primed for release. Moreover, as previously observed both in hippocampus and prefrontal/frontal cortex [11,14,19], the 15 mM KCl-induced overflow of GABA was not modified by venlafaxine or agomelatine (Figure 1A).

### Chronic antidepressant treatments reduce SNARE complex in hippocampal presynaptic membranes

The core of the presynaptic SNARE complex is formed by the coiled-coil domain interaction of two membrane proteins (syntaxin-1 and Soluble NSF Attachment Protein-25, SNAP-25) and one vesicle protein (synaptobrevin-2) [20]. A large body of evidence demonstrated that the SNARE complex mediates the fusion of synaptic vesicles and neurotransmitter release [21,22].

Because in hippocampus glutamate synaptic terminals represent the vast majority of total synapses [4], molecular changes in the presynaptic release apparatus can be likely correlated with changes in glutamate release. Thus, we evaluated if the decrease of glutamate release induced by agomelatine and venlafaxine was associated with altered assembling of presynaptic SNARE complexes.

The analysis of SNARE complexes in purified presynaptic membranes revealed two major syntaxin-1-containing complexes, migrating at ~100 kDa and ~80 kDa (Figure 1B), as previously reported [14]. No significant changes were found in total syntaxin-1 levels (Figure 1C). In line with the reduction of depolarization-dependent glutamate release, chronic treatment with both venlafaxine and agomelatine significantly reduced the accumulation of 80 kDa SNARE complex in synaptic membranes (F_2,25_ = 5.306; p < 0.05; -23.47% and -19.17% for venlafaxine and agomelatine respectively) (Figure 1D). No significant changes were found in 100 kDa SNARE complex accumulation (Figure 1E).

Overall, the present findings show that chronic administration of agomelatine or the reference antidepressant venlafaxine significantly reduced the endogenous release of glutamate from hippocampal synaptosomes, as previously found with other traditional antidepressants [11]. At the same time, both drugs decreased the accumulation of SNARE complexes in presynaptic membranes.

## Discussion and conclusion

This work demonstrates that chronic treatment with the new antidepressant agomelatine, a MT1 and MT2 receptor agonist and 5-HT_2C_ receptor antagonist, as well as with venlafaxine, a selective serotonin and noradrenaline reuptake inhibitor, markedly reduces depolarization-evoked endogenous release of glutamate from hippocampal synaptosomes. Interestingly, the depolarization-evoked release of GABA was not modified, suggesting that both drugs selectively inhibit glutamatergic release without affecting GABA transmission. These results are in line with previous studies, both in vivo and ex vivo, showing similar effects on glutamatergic transmission of a number of traditional antidepressants and of agomelatine ([23,24]; discussed and reviewed in Ref. [3] and [10]).

The reduction of glutamate release with no changes in GABA release suggests an alteration in the balance between excitatory and inhibitory neurotransmission that could improve the signal to noise ratio in glutamate transmission, when it becomes compromised by excessive release. In this regard, we have demonstrated that chronic treatment with different classes of antidepressants, includ-ing agomelatine, was able to completely prevent the marked increase of depolarization-evoked glutamate release from prefrontal and frontal cortex synaptosomes induced by acute stress [14,19].

Another intriguing result of this study is that, despite chronic agomelatine inhibited glutamate release evoked by 15 mM KCl depolarization, it had no effect on release induced by 0.5 mM ionomycin. Indeed, if it is generally agreed that electrical/chemical depolarization mainly induces the fusion of vesicles of the readily releasable pool (RRP) [25], ionomycin also promotes calcium-dependent fusion of vesicles, but mainly from the reserve pool. Therefore, our results suggest that agomelatine may selectively affect the RRP of vesicles, thereby altering a physiologically relevant pool for neurotransmitter release. In line with this hypothesis, we found that the reduction of glutamate release induced by chronic agomelatine and venlafaxine was accompanied by reduced accumulation of SNARE complexes in synaptic membranes (the fraction of synaptosomes containing the RRP). These data are in line with our previous results showing that traditional antidepressant-induced reduction of glutamate release is accounted for by changes in protein-protein interactions regulating the assembly of the SNARE complex [11], and by reduction of complex accumulation in presynaptic membranes [13]. However, contrary to previous results with traditional antidepressants (2 weeks of treatment) [11], here we did not find major changes in the expression of syntaxin-1 after 3 weeks of treatment with agomelatine or venlafaxine. Because the same result was obtained with both drugs, we suggest that the difference from other antidepressants is probably due to the different timing of drug-treatment (3 vs 2 weeks). However, the main finding is that, after treatment for 3 weeks, agomelatine and venlafaxine still reduced both the accumulation of SNARE complexes at the level of the RRP and the physiological release of glutamate.

Indeed, the SNARE complex and associated proteins play a critical role in vesicle docking, priming, fusion and synchronization of neurotransmitter release at presynaptic membranes and it was established that the SNARE complex corresponds to the minimal machinery for membrane fusion in eukaryotic cells, forming a stable complex that make the vesicles competent for fusion [21,22,26]. Therefore, a reduction of SNARE complex accumulation in synaptic membranes is consistent with reduced neurotransmitter release.

In conclusion, the present study demonstrated that chronic agomelatine dampens hippocampal glutamate neurotransmission, a likely component of the therapeutic action of antidepressants. The intriguing finding of reduced accumulation of SNARE complexes in presynaptic membranes suggests selected mechanisms in the exocytotic machinery as possible molecular targets of these drugs.

## Methods

### Animals and treatments

Adult male Sprague–Dawley rats (170–200 g) were purchased from Charles River (Calco, Italy). Animals were kept at constant temperature (22°C) with a regular 12 h light/dark cycle (light-off at 7 pm). The rats were housed in groups of four with ad libitum access to food and water. Rats were treated with agomelatine (40 mg/kg i.p.), venlafaxine (10 mg/kg i.p.) or vehicle (hidroxyethylcellulose, 1%, 1 ml/Kg, i.p.) for 21 days. All drugs were administered at 5.00 pm (2 h before the start of the dark cycle, 7 pm). Animals were sacrified 16 h after the last drug administration, and the hippocampus (HPC) was quickly removed.

All animal procedures were conducted according to current regulations for animal experimentation in Italy (Decreto Legislativo 116/1992) and the European Union (European Communities Council Directive 86/609/EEC) and following authorization by the Italian Ministry of Health to the University of Milano for experimental use of animals (Decreto Legislativo 295/2012-A).

### Preparation of purified synaptosomes

Purified synaptic terminals (synaptosomes) were prepared by centrifugation on Percoll gradients [27], with minor modifications as previously described [14,28]. Purity of synaptosomes and other subcellular fractions were evaluated by electron microscopy and by measuring subcellular distribution of protein markers (not shown), as previously reported [29].

When used for neurotransmitter release experiments, synaptosomes were resuspended in physiological medium with the following composition: 140 mM NaCl, 3 mM KCl, 1.2 mM MgSO_4_, 1.2 mM CaCl_2_, 1.2 mM NaH_2_PO_4_, 5 mM NaHCO_3_, 10 mM glucose, 10 mM HEPES, pH 7.2–7.4. For western blotting experiments, synaptosomes were resuspended in lysis buffer: 120 mM NaCl, 20 mM HEPES pH 7.4, 0.1 mM EGTA, 0.1 mM DTT, containing 20 mM NaF, 5 mM Na_2_PO_4_, 1 mM Na_2_VO_4_, and 2 mg/ml of protease inhibitor cocktail (Sigma-Aldrich, Milan, Italy). Further fractionation of freshly purified synaptosomes into synaptic membranes was carried out by differential centrifugation as previously reported [14].

### Endogenous glutamate and GABA release experiments

Endogenous neurotransmitter release was measured as previously reported [19,30]. Synaptosomes (about 100 μg of protein) were layered on microporous filters at the bottom of a set of parallel superfusion chambers maintained at 37°C [30]. Superfusion was started at a rate of 0.5 ml/min with standard medium. After 36 min of superfusion, samples were collected according to the following scheme: two 3-min samples (t = 36–39 min and t = 45–48 min; basal outflow) before and after one 6-min sample (t = 39–45 min; stimulus-evoked release). A 90-sec period of stimulation was applied at t = 39 min, after the first sample has been collected. Stimulation of synaptosomes was performed with 0.5 μM ionomycin or 15 mM KCl, the latter substituting for an equimolar concentration of NaCl.

Fractions collected were analyzed for endogenous glutamate and GABA content. Endogenous glutamate and GABA were measured by high performance liquid chromatography analysis. Amino acid release was expressed as nmol/mg of protein. The stimulus-evoked overflow was estimated by subtracting transmitter content of the two 3-min samples (basal outflow) from release evoked in the 6-min sample collected during and after the depolarization pulse (stimulus-evoked release). Drug treatment effects were evaluated by comparing the stimulus-evoked overflow in drug-treated animals vs. that calculated in vehicle treated rats. Appropriate controls were always run in parallel.

### Measurement of SNARE complex in isolated presynaptic membranes

For detection of SDS-resistant SNARE complexes, Western blotting was performed on samples of electrophoresed presynaptic membranes (non-boiled before gel loading) [14], incubating PVDF membranes containing blotted proteins with monoclonal antibodies for syntaxin-1 1:5000 (Sigma-Aldrich). The membranes were incubated with anti-mouse secondary antibody 1:4000 (Sigma-Aldrich), and immunoreactive bands revealed with ECL™ (GE Healthcare, Italy). Membranes were immediately exposed to Hyperfilm ECL™ films (GE Healthcare), and images acquired with the Quantity One software and GelDoc imaging system (Bio-Rad Laboratories, Italy). All bands used were within linear range of standard curves, and normalized for syntaxin-1 monomer levels in the same membrane. Standardization and quantitation of digitalized images were performed with Quantity One software (Bio-Rad).

### Statistical analysis

Statistical analysis was carried out by one-way analysis of variance (ANOVA) followed by Newman-Keuls post-hoc test. Significance was assumed at p < 0.05. Statistical analysis of the data was carried out using GraphPad Prism4 (GraphPad Software Inc., USA). Data are expressed as mean ± SEM.

## Abbreviations

ANOVA: Analysis of variance; GABA: γ-Amminobutyric acid; MT1: MT2, Melatonergic receptor1 and 2; RRP: Readily releasable pool; SNAP-25: Soluble NSF Attachment Protein-25; SNARE: Soluble N-ethylmaleimide-sensitive fusion protein attachment protein receptor.

## Competing interests

MP received research support and/or has been consultant for: Abiogen, GlaxoSmithKline, Merck Sharp & Dohme, Abbott, Servier, Fidia. GR has scientific collaborations with and is a member of the scientific board for Eli Lilly, Innova Pharma, and Servier. EM and CG are full-time employees of Institut de Recherches Internationales Servier (I.R.I.S.). MM, DT, LM, GT, AM, TB and GB: nothing to disclose.

## Authors’ contributions

MP, GB and GR designed the study. EM and CG participated on study design and paper discussion. DT, LM and MP wrote the ms. MM, AM, GT, TB performed the experimental work. DT, MM and LM performed the statistical analysis. All authors read and approved the final manuscript.
